# Impact of oxytetracycline exposure on the digestive system microbiota of *Daphnia magna*

**DOI:** 10.1371/journal.pone.0265944

**Published:** 2022-04-27

**Authors:** Sarah B. Lovern, Rochelle Van Hart

**Affiliations:** Department of Life and Earth Science, Concordia University Wisconsin, Mequon, WI, United States of America; VIT University, INDIA

## Abstract

Antibiotics are used to treat serious illness, but may also be used extraneously or as a preventative measure in many farm animals. This usage increases the potential for unintentional exposure to a variety of organisms. When antibiotics enter aquatic environments, *Daphnia magna* are especially vulnerable as they filter-feed in freshwater environments. Oxytetracycline (OTC) is a commonly-used broad-spectrum antibiotic used to treat a variety of mammalian diseases. In this study, the impact of OTC on *D*. *magna* mortality and gut biota were studied using both cultivation and sequencing-based approaches. Mortality rates were extremely low with the LD50 >2,000ppm. However, OTC impacted abundance and species diversity of intestinal microorganisms in the gut of the *D*. *magna* in abundance as well as species diversity. In control organisms, *Pseudomonas putida* and *Aeromonas hydrophila* were both present while only *P*. *putida* was found in OTC-exposed organisms. Disruption of the intestinal biota in *D*. *magna* could have implications on long-term survival, energy expenditure, and reproduction.

## Introduction

Antibiotic therapy is crucial to treat life-threatening infections, but misuse of antibiotics leads to resistance in common pathogens [1]. Nevertheless, many animal herds are dosed with various antibiotics via injection or consumption within dietary feed. Since the 1970s, antibiotic supplements have been used and are still used to promote growth, increase performance, or help maintain general animal health in cattle feedlot operations [2–4]. With the standard use of antibiotic treatments for large groups of farm animals, inadvertent exposure of other organisms within these environments can occur and cause negative impacts.

Large corporate farms realize the danger of housing a great population of animals in close proximity. According to Compassion in World Farming [5], stressful conditions and crowding in farm animals promote disease while hindering health. Spread of contagious disease is a threat to animal health and productivity [6] so administration of an antibiotic can be an effective tool to prevent the spread of disease. Commonly administered to aid the health of many population is the broad-spectrum antibiotic oxytetracycline (OTC). OTC is used to treat a variety of infections in humans, cattle, sheep, and pigs [7]. For example, Muuka et al. [8] studied a herd of 500 cattle that experienced pneumonia. Sixty-eight animals were treated with OTC and resulted in twenty-nine surviving (42.6%) [8]. Conversely, antibiotics are often used as a deterrent to disease in otherwise healthy organisms. For example, OTC is also injected into cattle to prevent anaplasmosis [9, 10], not as a result of an actual infection.

Incorporation of antibiotics into animal feed as a preventative measure exacerbates their presence in freshwater sources as well. Farms release massive quantities of pollutants through runoff from livestock operations [11]. Studies have shown that partial absorption appears to apply to ingested antibiotics used in animal feed Almost fifty years ago, Elmund et al. [2] found that 75% of dietary chlortetracycline was excreted in manure from yearling steers. Presently, commonly-administered antibiotics like OTC and its metabolites are also found in the excrement of cattle [12]. The use of antibiotics in livestock farming is also a worldwide problem. For instance, widespread antibiotic usage in Cambodia was found to be propagated by four factors: belief that antibiotics were necessary, limited knowledge among farmers, unrestricted access to pharmaceuticals, and weak monitoring of their administration [13]. This prolific usage of antibiotics has resulted in levels of OTC found in the Weihe River in China ranging from 1.56 to 87.89 ng/L, [14], 100 ng/L in Australia [15], and 340 ng/L in the United States [16]. Additionally, the half-life of OTC in water at 25°C, pH 7.0 has been shown to be 14.04 ± 5.41 days [17].

In recent years, the introduction of pharmaceuticals into freshwater ecosystems has become a large concern. Modern, technological advances have enabled the detection of various pharmaceuticals in water around the globe [18]. These pharmaceuticals can be introduced to our freshwater environments through agricultural waste, poor disposal of unused prescriptions, and active metabolites expelled through fecal matter. They have been found in water, sediments, and within biota in levels that are capable of causing detrimental effects on aquatic organisms [19]. Fifty percent of a dosage of OTC in mammals will not be absorbed, but instead pass through the digestive tract and remain unmetabolized within fecal matter [20]. These compounds are then released from fecal matter and are found in freshwater. Conventional wastewater treatment methods have been found inadequate in the removal both pharmaceuticals and their active metabolites [21]. Wastewater treatment plants (WWTPs) still often use the activated sludge process as it is an acceptable, low-cost way to produce wastewater effluent; however, this treatment’s ability to remove pharmaceuticals is limited [22] and tetracycline antibiotics can be detected even after treatment at wastewater facilities [23]. OTC was detected at 19.5 ± 2.9 mg/L in treated outflow (effluent) of a wastewater treatment facility [24]. Additionally, the concentrations of various pharmaceutically active compounds in discharged effluents are highly variable [25] due to seasonal variations or increased storm water runoff. Limited knowledge of the toxicological implications of chronic exposure to a variety of pharmaceuticals at sub-therapeutic levels to non-target organisms exists [19].

Organisms useful in the examination of antibiotic exposure into aquatic environments are *Daphnia magna* as they occupy a critical position in the food web [26] and are a standard species for toxicology testing [27]. *D*. *magna* are filter feeders that consume small particles such as bacteria, algae, rotifers, and copepod nauplii- for nutrition [28]. One individual *D*. *magna* which is only 2–3 millimeters in length will filter nearly 400 millilters of water each day [29]. *D*. *magna* are important to the aquatic environments as primary consumers grazing on algae and providing food for juvenile fish [28]. Because *D*. *magna* have such significant contact with their surrounding environment on a daily basis, these organisms are especially susceptible to contaminants in water. Exposure to an antibiotic could impact *D*. *magna* in a variety of ways including gut microbiota. The microbial make up of an organism’s gut have been show to promote digestion, increase nutrient availability, detoxifying harmful compounds, and compete with pathogens [30]. As *D*. *magna* are a vital link in the food web, unintended exposure to antibiotics may potentially impact resources that many humans depend upon.

With their important role in the ecosystem, a concern arises about any adverse effects that pharmaceuticals may have on *D*. *magna* populations. As over 67 billion livestock were reared worldwide for meat in 2016 [31], administration of OTC and its entrance into the environment via excreted waste from these organisms, *D*. *magna* may be exposed to this drug. Antibiotics may affect nontarget organisms by changing their microbiota which could lead to compromised nutrition and therefore growth [32]. In order to recognize potential negative interactions, we investigated the relationship between *D*. *magna* and an antibiotic used in agriculture.

The presence of antibiotics in lakes and rivers can present a problem for the *D*. *magna* population. This study therefore examined the impact of low-dose exposure of oxytetracycline on *D*. *magna’s* mortality and gut biota population. Additionally, overuse of antibiotics can lead to resistance. Antibiotic resistance can make bacterial infections harder to control leading to more severe impacts on humans including longer illness duration as well as increased hospitalization and death [5].

## Materials and methods

Forty-eight hour acute toxicity tests were conducted with *D*. *magna* using U.S. EPA standard operating procedure 2024 [27] at Concordia University Wisconsin (43.2542°, 87.9155°). No permits were required by the CUW IACUC because these organisms are invertebrates. *D*. magna were obtained two years prior to the beginning of the experiment from both Carolina Biological and Ebay and combined in growth chambers. Organisms were cultured according to Lovern and Klaper [33] but were kept at 21°C and cultures were maintained in well water sampled from the Pryor Street Well in Milwaukee, WI, USA (pH 7.5–8.0). During prior behavior and reproduction trials in our lab (unpublished), mortality of *D*. *magna* was exhibited when exposed to OTC and not seen in control groups. Therefore, this study was conducted to examine mortality and compare it to established values [34]. Multiple trials were run at 40, 200, 400, 2000, 4000, 8000ppm to determine the LD50 and attempt to achieve 100% mortality.

To examine the impact of oxytetracycline exposure on gut biota, exposure trials were run for 72 hours. For each trial, a total of three replicates containing twenty *D*. *magna* in 200ppm of oxytetracycline and three replicates in control water were performed. OTC is widely found in aquatic systems at the μg/L level [23]. The larger dosage for this study was chosen to simulate a potential pulse exposure level found at the mg/L (ppm) level in China [35]. While this dosage is very high and unlikely to occur for long periods in natural environments, it allowed for acquisition of initial information on which gut flora species were impacted. In order to obtain a large intestinal region, *D*. *magna* selected for each trial ranged from two to four weeks in age. Experiments were conducted in 50 mL of solution and animals. Each container was fed *Chlamydomonas reinhardtii* and alfalfa at the start of the experiment and once more at twenty-four hours. At 72 hours of exposure, the intestinal tracts of the organisms of ten organisms from each vessel were removed via microdissection and placed in PBS buffer. The samples were then stored on ice. This experiment was repeated four times with the same number of digestive tracts sampled each occasion (n = 40 experimental tracts and 40 control tracts.)

The contents of the intestinal tracts of the D. magna were then sonicated for 30 seconds. The solutions were then triturated with a sterile micropipette to ensure an evenly mixed solution. Serial dilutions ranging from 1 x 10–1 to 1 x 10–6 were plated on tryptic soy agar and incubated at 37⁰C for 20 hours. The bacterial growth on the plates was then observed and the bacterial colonies were counted. Samples of each unique morphological colony were selected from the growth plates after 20 hours. These samples were then gram-stained, sent to Genewiz, Inc. [36] for 16s rRNA sequencing and analyzed using The Basic Local Alignment Search Tool (BLAST). Results yielded species identification rates of 96–99%.

## Results

**Fig 1:**
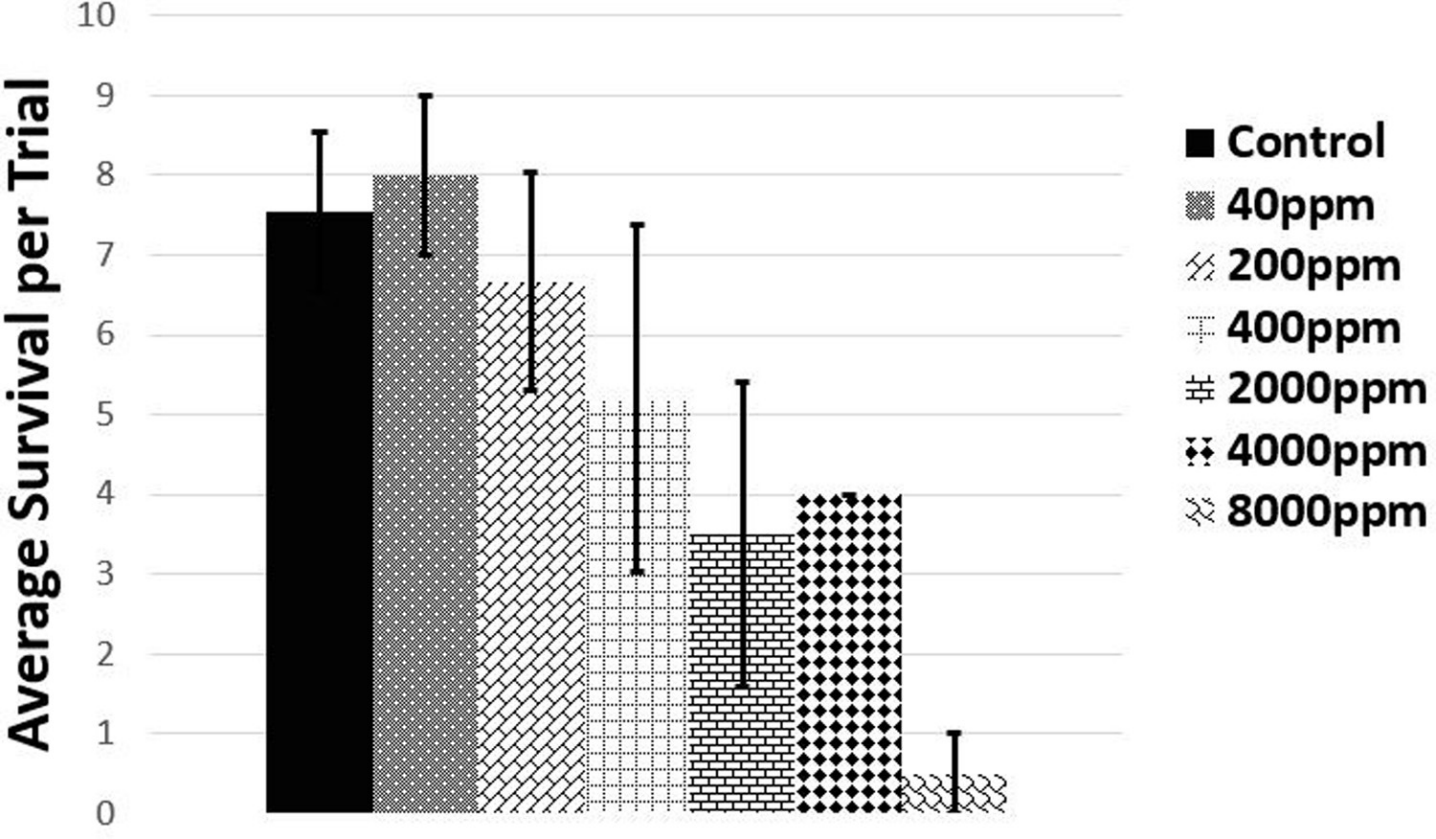
Forty-eight day trials were conducted to find the LD50 value for exposure to oxytetracycline. Average survival (arithmetic mean) of *D*. *magna* for each concentration of OTC are shown on the image. Each trial contained ten *D*. *magna* with four replicates for each concentration. Error bars represent one standard deviation.

Mortality ranged from zero within control water to 100% in one of the 8000ppm trials (Fig 1). However, one-hundred percent mortality during each 8000ppm trial was not obtained but resulted in an average of 90% lethality. As this is not a biologically-relevant concentration, an increase in drug concentration to obtain 100% mortality was discontinued. Using the AAT Bioquest LD50 calculator [37], the LD50 was determined to be >2,000ppm (mg/kg). This is comparably high as others have found it to be 6696mg/kg in a mouse [38] and oxytetracycline has been classified as practically non-toxic to *D*. *magna* [34].

Initial stomach contents were Gram stained and revealed gram-negative rods. Intestinal content was then further analyzed and revealed an increase in colony forming units per milliliter (CFU/ml) in the gut of *D*. *magna* exposed to Oxytetracycline (3.3 x 10^5^ CFU/ml; SD = 881, SEM = 509) as compared to control organisms (9.0 x 10^3^ CFU/ml; SD = 2.5 x 10^3^, SEM = 1.47 x 10^3^). The result of over four times as many bacteria being present in the guts of animals exposed to the antibiotic was initially deemed perplexing. Furthermore, variations in colony morphology were observed between treated and control Daphnia, indicating a difference in resident bacterial species. Therefore, 16s rRNA sequencing [36] of bacterial colonies isolated from *D*. *magna* before and after exposure to oxytetracycline was conducted to determine gut flora composition. Sequence data for control isolates confirmed the presence of both *Pseudomonas putida* and *Aeromonas hydrophila*, whereas isolates from exposed *D*. *magna* were all *Pseudomonas putida*.

## Discussion

OTC seems to be disrupting the gut flora that were detected when cultured in the lab. This disturbance leads to opportunistic infection by *P*. *putida* and elimination of *A*. *hydrophila* from the gut tissue. The ability of *P*. *putida* to abound in the presence of OTC is of no surprise as *P*. *putida* was found as the most abundant bacterial group in wastewater from an OTC treatment facility [39].

This study used viable plate count to determine colonies, but we understand the limitations of this technique in detecting all bacteria present [40]. Jones (1977) showed that the viable count contained a mere 0.25% of the biodiversity found in total counts in pure freshwater [41], so we understand that additional species are likely found within the gut of *D*. *magna*. However, the impact of the microbiota of an organism is recognized as integral component of general health due to the contribution of these microorganisms to many biological processes [30] Therefore, finding a shift in the bacteria growth via viable plate count demonstrates a potential detriment to the health of *D*. *magna*.

Callens et. Al [42] showed that the composition of the bacterial community within the surrounding waters had a major influence on the *D*. *magna* gut bacterial community [42]. As OTC readily desorbs from the riverine sediment [43], OTC from metabolic waste can enter into aquatic systems. This release may then impact bacteria populations in ecosystems that then change bacterial availability. Health and fitness of many animal species can depend upon gut bacterial community composition [42].

The results of this study also indicates that the antibiotic is taken up in the feeding column and enters the gut tract of the *D*. *magna* during feeding. This was to be expected because OTC has a tendency to adsorb onto any particles in an aquatic environment. In previous research it was not always clear whether the agglomerated oxytetracycline was active or not [44]. The elimination of the *A*. *hydrophila* population shows that the OTC remained bioactive in the digestive tracts of the *D*. *magna*. This could mean that the main food source for any filter-feeding organism contains the pharmaceutical or that *D*. *magna* take in the antibiotic via feeding streams of liquid created by their generation of a feeding column [28]. OTC may also actively inhibit free-floating microbes in aquatic environments, thus disturbing the microbial food web.

In mammals, Han et al. [25] found that rats exposed to 4-epi-oxytetracycline (EOTC), the main product of OTC metabolism, showed an accumulation of the metabolite and altered intestinal microbiota. The relative abundance of Bifidobacteriaceae, a type of Actinobacteria, increased as well as an overall escalation in abundance of antibiotic resistance genes (ARGs) [25]. In addition to EOTC, Turker et al. [12] showed that alpha-apo-oxytetracycline and beta-apo-oxytetracycline are also found in manure of cattle injected with OTC [12]. Therefore, OTC and its metabolites may impact the gut biota of *D*. *magna* if they are exposed to this antibiotic.

Along with the implications of the intestinal tracts of small crustaceans in our freshwater, the introduction of antibiotics into the environment can contribute to other large problems. Just as chronic exposure to a toxicant can negatively impact *D*. *magna* survival, reproduction, and growth [45], extended exposure to antibiotics may also lead to decreased fecundity and viability. Exposure to trimethoprim has been found to decrease the abundance of bacterial species associated with *D*. *magna* to twenty times less that of the control exposure [32]. Additionally, the preventative use of antibiotics causes the release of antibiotic resistance genes (ARGs) into fecal waste [12] and these can reach aquatic ecosytems. Aquatic environments play an important part in the dispersal of antibiotic-resistant bacterial organisms [46].

The prophylactic use of antibiotics in feed and water of healthy animals is dangerous and unjustifiable [5]. The results of this study showed that the relatively non-toxic antibiotic OTC is impacting the distribution of species in the gut of the *D*. *magna*. The impact is not purely on the number of bacteria found but also on the types of species present. In the control organisms, *Pseudomonas putida* and *A*. *hydrophila* were both present while only *P*. *putida* was found in the organisms exposed to the OTC. However, abundance of total bacteria in the gut showed a greater number of organisms in the digestive tract of the exposed *D*. *magna*. This was counterintuitive to what was initially predicted since antibiotics function to inhibit the growth of bacteria. However, it appears that the antibiotic played a role in allowing for one species of bacteria, *P*. *putida*, to overtake the digestive environment with the weakening of *A*. *hydrophila*. Disruption of the control intestinal biota in *D*. *magna* may have implications on long-term survival, energy expenditure, and reproduction and should be further examined. Additionally, now that the impacted gut flora species have been determined, more realistic continuous dosages around 1.56 to 340 ng/L [14–16] can be studied to see what the short and long-term impacts on *D*. *magna* are. If the formation of laws and regulations as well as education of farmers worldwide can continue, this effort can be paired with good husbandry and hygiene that can often prevent disease spread instead of preventative antibiotic administration [5].
